# The Current Landscape of Congenital Heart Surgery in Northern China: A Geographic and Population-Based Analysis

**DOI:** 10.3389/fped.2021.555141

**Published:** 2021-05-07

**Authors:** Zhanhao Su, Li Xiang, Zeye Liu, Hao Wu, Shoujun Li, Huiwen Chen, Hao Zhang

**Affiliations:** ^1^State Key Laboratory of Cardiovascular Disease, National Center for Cardiovascular Diseases, Fuwai Hospital, Peking Union Medical College, Chinese Academy of Medical Sciences, Beijing, China; ^2^Center for Pediatric Cardiac Surgery, National Center for Cardiovascular Diseases, Peking Union Medical College, Fuwai Hospital, Chinese Academy of Medical Sciences, Beijing, China; ^3^Shanghai Children's Medical Center, National Children's Medical Center, Heart Center and Shanghai Institution of Pediatric Congenital Heart Diseases, Shanghai Jiaotong University School of Medicine, Shanghai, China

**Keywords:** congenital heart disease, congenital heart surgery, health disparities, access to care, China

## Abstract

**Background:** Congenital heart disease (CHD) is the leading birth defect in China, and many patients require congenital heart surgery (CHS) to achieve optimal outcomes. However, the current landscape and population accessibility to specialist resources for CHS in China are unclear.

**Methods:** Care facilities performing CHS were identified from the 2018 white book of Chinese cardiovascular surgery and were categorized as full or limited facilities based on specialist capacity. Census-based population data and road data were obtained from administrative sources. Service area analysis of all facilities was performed to estimate geographic accessibility.

**Results:** Of 93 facilities in 14 provinces and municipalities in northern China, only 30.1% had full specialist capacity. The shortage of pediatric cardiologists and mechanical circulatory support was the primary limiting factor. In 2018, 61.3% of facilities performed <200 CHS cases, and 31.6% of all CHS cases (*N* = 30,921) were performed in limited facilities with substantially lower volume than full-capacity facilities (median value: 85.0 vs. 368.0). Beijing had a disproportionately higher CHS volume (367 cases per million population) than other provinces. Of all children under 5 in northern China, only 12.9% live within 30 km (a typical half-day visit) of the service areas of all facilities. Compared to children from the eastern region (31.4%), 71.8% of children from the central region and 70.2% of children from the western region needed to travel >180 km (a typical overnight visit) to receive care in full-capacity facilities.

**Conclusions:** Many facilities for CHS in northern China had limited specialist capacity, and many CHD patients received suboptimal surgical care. Policy measures should address the significant geographic disparities to receive high-quality surgical care among disadvantaged patients.

## Introduction

Congenital heart disease (CHD) is a rising global health burden in affected children and adults. The recent analysis from the 2017 Global Burden of Disease study estimates that 12 million patients are living with CHD around the world, causing a total of nearly 600,000 years lived with disability and over 270,000 deaths (1). The treatment of CHD is typically surgical. Hence, patients' access to proper facilities with capacity for congenital heart surgery (CHS) is critical for survival and optimal outcomes. However, it is estimated that over 70% of the world population have limited or no access to cardiac surgical care, which creates substantial unmet needs in pediatric heart surgery (2).

As one of the most populous countries in the world, China is facing the challenge of treating a growing CHD population with relatively limited specialist resources for heart surgery. The burden of CHD in China could be higher than estimated because of inadequate healthcare resources (3). A recent report on cardiac surgical workforce showed that the number of registered adult and pediatric cardiac surgeons is only 0.1–0.5 per million population in China (4). In 2018, a global study assessing personal healthcare access and quality reported that the Healthcare Access and Quality (HAQ) index for CHD in China is substantially lower than the national HAQ index (36 vs. 78) (5), suggesting that many CHD-related deaths would have been preventable if every affected patient received proper cardiac care. Despite the significance of this issue, current literature lacks sufficient data to inform health policy and quality improvement initiatives for CHD care in China. Notably, the distribution, availability, and specialist capacity of treatment resources for CHS are largely unknown and remain to be elucidated in China.

Measuring the distribution of and geographic access to healthcare resources is important because delayed access to receive specialized care contributes to adverse outcomes among CHD patients. Indeed, a recent US nationwide analysis found that CHD-related infant mortality differs by mother's residential proximity to specialized pediatric heart centers (6). Newborns with hypoplastic left heart syndrome delivered at sites far from cardiac surgical centers have increased presurgical mortality (7). Furthermore, the negative impact of disadvantaged geographic factors can contribute to discontinuation of follow-up care after surgical treatment, as studies reported that longer distance and transit time to heart centers can substantially increase patient's travel burden during clinic visit (8, 9). Therefore, the research community has placed increasing focus on characterizing the geographic accessibility to care facilities for CHD. However, current studies on this topic published to date are mainly derived from resource-abundant regions (such as Europe and North America), whereas studies from developing countries with an emerging economy like China are very limited.

In this study, we provided the first analysis to characterize the geographic distributions and specialist capacity of care facilities for CHS in northern China. Using the Geographic Information System (GIS) technology, we identified profound disparities in geographic access to high-quality care between different economic regions. Our findings highlight the necessity of capacity building for many care facilities in order to address the rising burden of CHD in China, as well as the importance of reducing geographic disparities to receive care.

## Methods

### Overview of Study Design and Study Area

In this study, we included 14 provinces and municipalities in northern China, namely, Beijing, Tianjin, Henan, Hebei, Shandong, Liaoning, Jilin, Heilongjiang, Shanxi, Shaanxi, Qinghai, Gansu, Inner Mongolia, and the Ningxia Hui autonomous region. We focused on these provinces (covering 42.4% of the total land area and 40.2% of the total population in mainland China) rather than the whole country, because residents in these regions tend to have similar health-seeking behaviors due to geographic proximity and cultural factors. Provinces in China are grouped into eastern, central, and western regions based on their socioeconomic development status according to the *China Statistics Year Book*. Generally, provinces in the eastern region are more economically developed and have more healthcare resources than provinces in central and western regions (10).

### Identification and Evaluation of Care Facilities Performing CHS in Northern China

The Chinese Society of Extracorporeal Circulation (ChSECC) is currently the only academic society in China with a registered nationwide database for cardiac surgery (11). There are 282 facilities in northern China participating in the registration. For the purpose of this study, we screened and included facilities performing CHS in 2018 and collected data on the total volume of CHS and the cardiopulmonary bypass (CPB) volume under 18 years old. Facilities with missing data on pediatric cases or without pediatric cases were excluded. Data on general characteristics of these facilities were collected with a predefined data collection form, including location, hospital type (general hospital, children's hospital, or specialized hospital), ownership, and teaching status. To evaluate the specialist capacity of these facilities, we adopted the criteria published by the American Academy of Pediatrics (12), and we collected information on the following items: non-invasive imaging modalities, intensive care, pediatric cardiologist, cardiac catheterization, electrophysiology, and mechanical circulatory support. All hospital-level data were extracted from official hospital websites.

The identified facilities are categorized into two groups based on specialist capacity. Care facilities with full specialist capacity are able to perform CHS, have at least one staffed pediatric cardiologist, have advanced imaging modalities for non-invasive diagnosis or evaluation, have cardiac catheterization and electrophysiology service, and have mechanical circulatory support. Care facilities with limited specialist capacity are defined as being able to perform CHS but not able to meet all the criteria listed for full capacity (Table 1).

**Table 1:** Categorization of care facilities for CHS.

	**Full** ** capacity**	**Limited capacity**
CHS		
Practicing surgeon with pediatric surgical expertise	Yes	Yes
Anesthesiologist with pediatric expertise	Yes	Yes
Cardiovascular intensive care	Yes	Yes
Non-invasive imaging modalities		
Echocardiography	Yes	Yes/no
Cardiac MRI	Yes	Yes/no
Cardiac CT	Yes	Yes/no
Pediatric cardiologist	Yes	Yes/no
Cardiac catheterization	Yes	Yes/no
Electrophysiology	Yes	Yes/no
Mechanical circulatory support	Yes	Yes/no

*MRI, magnetic resonance imaging; CT, computed tomography; CHS, congenital heart surgery*.

*Limited capacity is defined as programs capable of performing pediatric cardiac surgery but not meeting all the criteria of full capacity*.

### Calculation of CHS per Million Population and CHD Burden in Live Births

Census-based population data were accessed through the NASA Socioeconomic Data and Application Center (SEDAC) (13). This SEDAC dataset was based on the 2010 national census of China and provided an estimated number of populations in 2015 within each population centroid. The population centroid is the smallest administrative unit in China (“Jiedao,” i.e., streets clustered as a local community). To calculate the number of CHS per million population in different regions, the SEDAC dataset was spatially joined with the facility dataset, and CHS cases were aggregated at the provincial level.

Since 1996, the Chinese government has established a nationwide surveillance network to monitor the incidence of birth defects in newborns. For the purpose of this study, we obtained data on the incidence of CHD among live births in each province in 2014 from the China Maternal and Child Health Surveillance System (14). The number of live births in each province was obtained from the *China Health Statistics Year Book* (http://www.stats.gov.cn/tjsj/ndsj/). The number of newly diagnosed CHD cases was estimated by multiplying live birth data and CHD incidence in each province.

### Geodata Sources and Service Area Analysis

Geographic accessibility to healthcare facilities can be measured by service area analysis. To perform service area analysis for identified facilities in this study, the location of each facility was first geocoded using XGeocoding version 2.0 and was mapped in ArcGIS version 10.6. The road network and administrative boundary data of China were downloaded from the Topographic Database of the National Fundamental Geographic Information System of National Geomatics Center of China (http://www.webmap.cn/main.do?method=index) to construct a road network dataset in ArcGIS. The Network Analyst Extension in ArcGIS was applied to perform service area analysis for each care facility. Driving distance was selected as a surrogate for measuring travel impedance (travel cost) and has been used in previous geographic analysis in health service research (15). Breakdowns of driving distance indirectly reflect the travel time needed to attend a visit at a care facility: <30 km suggests a visit within half a day, 30–90 km suggests a visit within 1 day, 90–180 km suggests a trip likely necessitating an overnight stay, and >180 km generally requires at least one overnight stay. Service area analysis created multiple polygons based on predefined driving distance breaks, and the population centroids within these polygons were aggregated to calculate the percentage of population under different levels of geographic accessibility of care facilities for CHS.

### Statistical Analysis

Characteristics of care facilities were summarized with descriptive statistics. Categorical variables were expressed as counts and percentage. Continuous variables were expressed as median with interquartile range (IQR). All analysis was performed in R (version 3.6.1).

## Results

The basic characteristics of care facilities for CHS in northern China were shown in Table 2. Of 93 facilities identified, the majority were located in general (72.0%), public (90.3%), and teaching (92.5%) hospitals. However, only 30.1% (*n* = 28) had full specialist capacity. Among the listed criteria of eligible facilities for congenital cardiac care, pediatric cardiologists and mechanical circulatory support were the primary limiting factors, and they were present in only 40.9% (*n* = 38) and 54.8% (*n* = 51) of all facilities. In facilities with limited capacity, only 16.9% had staffed pediatric cardiologist, and 35.4% were capable of mechanical circulatory support. The eastern region had a higher percentage of facilities with full capacity (64.3%), whereas the central and western regions were notable for higher percentages of facilities with limited capacity (43.1 and 21.5%). The median CHS volume differed substantially between full and limited facilities (368.0 vs. 85.0). Among facilities with full capacity, 85.8% had a CHS volume of >200 cases; by contrast, 81.5% of facilities with limited capacity had a CHS volume of <200 cases.

**Table 2:** Characteristics of care facilities for CHS in northern China.

	**Full capacity**	**Limited capacity**	**Overall**
Total (n)	28	65	93
Economic region, *n* (%)			
Eastern	18 (64.3)	23 (35.4)	41 (44.1)
Central	3 (10.7)	28 (43.1)	31 (33.3)
Western	7 (25.0)	14 (21.5)	21 (22.6)
Hospital type, *n* (%)			
General	18 (64.3)	49 (75.4)	67 (72.0)
Children	8 (28.6)	9 (13.8)	17 (18.3)
Specialized	2 (7.1)	7 (10.8)	9 (9.7)
Ownership, *n* (%)			
Public	25 (89.3)	59 (90.8)	84 (90.3)
Private	0 (0.0)	2 (3.1)	2 (2.2)
Military	3 (10.7)	4 (6.2)	7 (7.5)
Teaching hospital, *n* (%)	28 (100.0)	58 (89.2)	86 (92.5)
Cardiac catheterization, *n* (%)	28 (100.0)	58 (89.2)	86 (92.5)
Electrophysiology, *n* (%)	28 (100.0)	62 (95.4)	90 (96.8)
Pediatric cardiologist, *n* (%)	28 (100.0)	11 (16.9)	38 (40.9)
Mechanical circulatory support, *n* (%)	28 (100.0)	23 (35.4)	51 (54.8)
Total CHS volume (median, IQR)	368.0 (219.0, 873.5)	85.0 (50.0, 160.0)	129.0 (61.0, 323.0)
CPB cases <18 years (median, IQR)	239.5 (169.5, 817.5)	35.0 (11.0, 79.0)	61.0 (26.0, 201.0)
Total CHS volume category, *n* (%)			
<200	4 (14.3)	53 (81.5)	57 (61.3)
200–400	12 (42.9)	9 (13.8)	21 (22.6)
>400	12 (42.9)	3 (4.6)	15 (16.1)

*CHS, Congenital heart surgery; CPB, cardiopulmonary bypass; IQR, Interquartile range*.

The CHS volume stratified by facility characteristics and economical regions is shown in Table 3. In 2018, a total of 30,921 CHS cases were performed in 93 facilities. Almost two thirds (21,147/30,921, 68.4%) were performed in facilities with full capacity, with the remaining 31.6% performed in facilities with limited capacity. Among all CHS cases, 15.7%, 31.6%, and 27.9% were performed in facilities with low surgical volume (<200 cases), with limited capacity, and without pediatric cardiologists, respectively. The eastern region had a higher proportion of CHS performed in facilities with full capacity (86.9 vs. 39.6% and 61.1%), with higher surgical volume (69.6 vs. 53.3% and 66.1%), and with pediatric cardiologist (87.2 vs. 49.7% and 64.7%) than central and western regions, respectively.

**Table 3:** Congenital heart surgery volume stratified by facility characteristics and economical regions.

	**Number of CHS cases (%)**			
**Capacity category**	**Eastern region**	**Central region**	**Western region**	**Total**
Limited capacity	2,066 (13.1)	5,103 (60.4)	2,605 (38.9)	9,774 (31.6)
Full capacity	13,715 (86.9)	3,340 (39.6)	4,092 (61.1)	21,147 (68.4)
**Volume category**				
Low volume (<200 cases)	2,046 (13.0)	1,602 (19.0)	1,204 (18.0)	4,852 (15.7)
Medium volume (200–400 cases)	2,747 (17.4)	2,342 (27.7)	1,065 (15.9)	6,154 (19.9)
High volume (≥400 cases)	10,988 (69.6)	4,499 (53.3)	4,428 (66.1)	19,915 (64.4)
**Pediatric cardiologist**				
No	2,022 (12.8)	4,246 (50.3)	2,361 (35.3)	8,629 (27.9)
Yes	13,759 (87.2)	4,197 (49.7)	4,336 (64.7)	22,292 (72.1)

**Figure 1:**
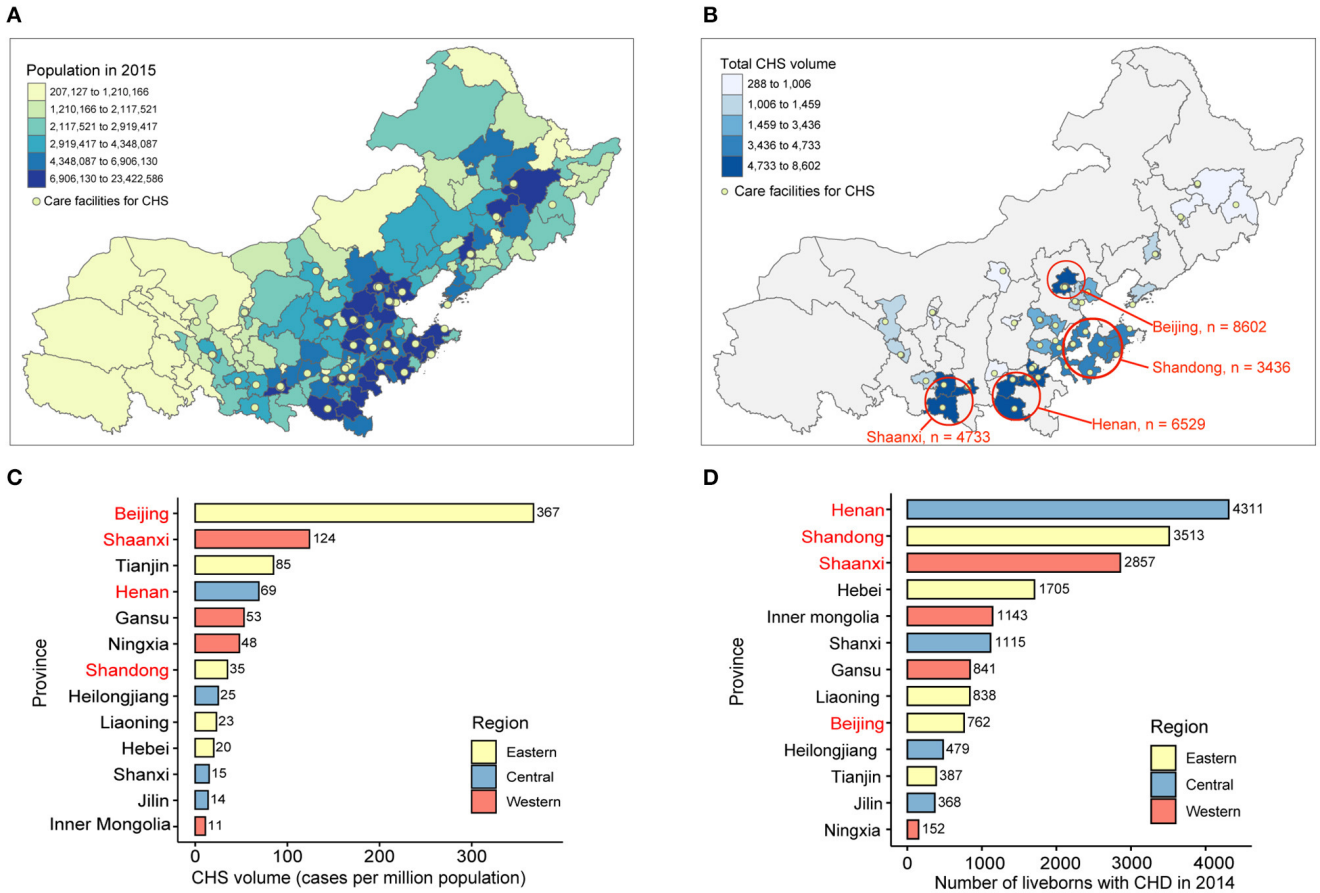
Geographic distribution of CHS volume and CHD burden in northern China. **(A)** Geographic distribution of care facilities for CHS. Population data are shown in quantiles. **(B)** Regional distribution of total CHS volume in northern China. The results of top four provinces are displayed in the map. CHS volume data are shown in quantiles. **(C)** Average CHS volume in million population, as calculated by the total CHS volume divided by total population in 2015 in each province. **(D)** Newly diagnosed cases of CHD in live births by geographic regions in 2014. CHS, congenital heart surgery; CHD, congenital heart disease.

Geographic mapping showed that care facilities for CHS were concentrated in regions with denser clusters of population, notably in the eastern and central regions (Figure 1A). In terms of total number of CHS cases, Beijing and Shandong (eastern region), Henan (central region), and Shaanxi (western region) were identified as the regional hubs for CHS (Figure 1B). However, Beijing has a disproportionately higher concentration of CHS volume (367 cases per million population) than other provinces (35, 69, and 124 cases per million population in Shandong, Henan, and Shaanxi, respectively, Figure 1C). The number of newly diagnosed CHD cases among live births in 2014 was highest in Henan (*n* = 4,311), Shandong (*n* = 3,513), and Shaanxi (*n* = 2,857), whereas Beijing had only 762 cases in 2014 (Figure 1D).

**Figure 2:**
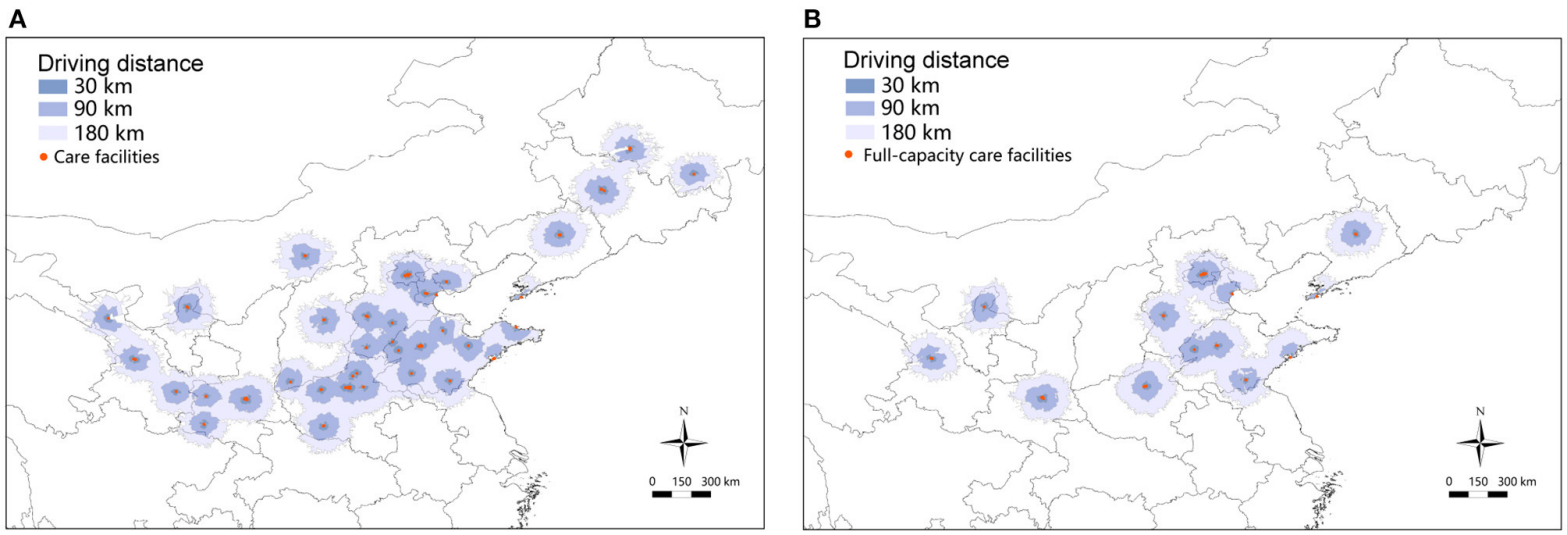
Service areas of facilities for CHS in northern China. **(A)** Service area of all care facilities for CHS. **(B)** Service area of full-capacity care facilities for CHS. The shaded area with different colors indicates different drive distance. A drive distance of <30 km suggests a typical half-day visit, <90 km a typical 1 day visit, <180 km a probable overnight visit, and >180 km suggests at least one overnight stay. CHS, congenital heart surgery.

The drive-distance-based service areas of identified facilities are shown in Figure 2. Of all children under 5 years old in northern China, only 12.9% live within 30 km (a typical half-day visit) of the service areas of all facilities. Compared with children from the eastern region (31.4%), the majority of children from the central and western regions (71.8 and 70.2%, respectively), needed to travel long distances (>180 km, a typical overnight visit) to reach full-capacity facilities to receive care (Table 4). When assessed for access to facilities with full capacity, there was a higher proportion of children from the central region (71.8%) and western region (70.2%), compared with those from the eastern region (31.4%), who needed to travel >180 km to reach facilities with full capacity (Table 4).

**Table 4:** Percentage of children under 5 within service areas of care facilities for congenital heart surgery in northern China.

	**Eastern region**	**Central region**	**Western region**	**Overall**
**Access to all care facilities**				
<30 km	15.2%	10.6%	12.1%	12.9%
<90 km	56.1%	36.7%	27.9%	43.7%
<180 km	87.3%	66.3%	47.6%	72.2%
>180 km	12.7%	33.7%	52.4%	27.8%
**Access to care facilities with full capacity**				
<30 km	9.3%	2.0%	7.5%	6.2%
<90 km	30.4%	8.5%	16.9%	19.6%
<180 km	68.6%	28.2%	29.8%	46.2%
>180 km	31.4%	71.8%	70.2%	53.8%

*A drive distance of <30 km suggests a typical half-day visit, <90 km a typical 1-day visit, <180 km a probable overnight visit, and >180 km at least one overnight stay. Therefore, different categories of drive distance can reflect different degrees of travel burden in different populations*.

## Discussion

Because of a large population base, China is facing a growing number of pediatric and adult CHD patients with limited specialist resources for cardiac surgery. Despite the importance of providing quality surgical care for these patients, the current landscape of CHS has not been elucidated in China. In this study, we are the first to characterize the geographic distribution and specialist capacity of care facilities performing CHS in northern China. Our study found that the majority of care facilities were providing suboptimal surgical care due to low surgical volume and the absence of pediatric cardiologists, and nearly one third of surgical patients were treated in these facilities with limited capacity. Moreover, we noted significant disparities in geographic access to receive care, with Beijing having a disproportionately higher concentration of CHS volume. Patients from central and western regions needed to travel substantially longer distances to receive high-quality care. Our study highlights that capacity strengthening and service improvement are urgently needed for many care facilities for CHS in China, particularly in the central and western regions.

In China, there is a documented trend of increasing CHD-related mortality. From 2003 to 2010, the overall CHD mortality rate increased from 141 to 229 per 10,000,000 person-years (16). Although the Chinese government had initiated a nationwide insurance scheme to reimburse treatment costs for children with CHD (17), many patients are having difficulty to access high-quality CHD care for various reasons. In this study, we found that nearly 5,000 cases of CHS (15.7%) in northern China were performed in facilities with a surgical volume of <200 cases. Furthermore, nearly 30% of all CHS were performed in facilities without pediatric cardiologists involved in routine care. Notably, these cases were mostly concentrated in the central and western regions. The association between surgical volume and surgical outcomes of CHS has been extensively reported in previous literature (18, 19), and the current guideline from Europe upholds that congenital heart surgeons should perform at least 150 cases per year to maintain the quality of care (20). Receipt of surgical treatment at lower-volume facilities is associated with higher rates of morbidity and mortality and higher hospitalization costs (21). Regionalization of care at higher-volume centers can improve the surgical outcomes of CHD patients, and therefore, it should be considered when designing a more effective treatment network of CHD in China.

High-quality programs in performing CHS are praised for their high cost-effectiveness, due to the potential healthy life gained in operated survivors and subsequent economic gain for the society (22). Therefore, our finding that many care facilities for CHS had suboptimal specialist capacity is concerning. We found that only 13% of care facilities performing CHS in northern China were staffed with pediatric cardiologists, which indicates the substantial shortage of pediatric cardiology workforce and is reflective of the nationwide shortage of pediatricians in China. According to the latest survey in 2016, the number of practicing pediatricians was only 4 per 10,000 children in mainland China, and the geographic distribution of pediatricians was extremely skewed toward the eastern region and metropolitan area (23). The shortage of pediatricians can affect the outcomes of CHD patients, as the absence of pediatricians in a surgical team could undermine the overall quality of care for the child because many CHD patients receive surgical treatment under 18 years old and pediatric cardiologists are trained to tailor the medical needs for this pediatric population. As the majority of CHD-related deaths occur under 1 year of age, training more cardiac physicians with pediatric expertise is critically important for improving the outcomes of CHD in China.

In this study, we identified an important pattern of CHS distribution in northern China. When CHS volume was viewed at the provincial level, Beijing (8,602 cases), Henan (6,529 cases), and Shaanxi (4,733) were identified as the regional centers for CHD treatment in eastern, central, and western regions of northern China, respectively (Figure 1B). However, when population and CHD burden data are taken into consideration, Beijing had a disproportionately higher concentration of CHS (367 cases per million population) than any other provinces. This finding strongly suggests that Beijing had attracted many CHD patients from other provinces to receive care, making itself the single largest regional hub in northern China. In the field of CHS, the concentration of volume in higher-level centers is indeed associated with lowered surgical mortality and complications (24), but it can substantially increase travel impedance as well as financial burden for patients traveling from disadvantaged regions to receive care, and it also contributes to variations in healthcare usage among patients from different regions. Indeed, a retrospective analysis of 525 patients undergoing total cavopulmonary connection surgery at Fuwai hospital shows that the median age at operation was 6.0 years (IQR: 4.2, 11), suggesting significant delays in receipt of surgical care for critical CHDs in Chinese patients (17). Moreover, disadvantaged geographic factors contribute to adverse outcomes of CHD, as studies report that CHD-related mortality was 28% higher among infants whose mother's residence was not proximal to specialized centers (6). Although treatment in higher-level centers is associated with better in-hospital outcomes, the disproportionate concentration of CHS volume in a few large cities could have adverse impact on the overall outcomes of CHD patients. Further studies are warranted to determine the association between disparities in geographic access to high-quality care and outcomes of CHD on a population scale.

In developed countries, there is an ongoing trend toward regionalization of CHS (25). However, for countries with limited healthcare resources like China, this idea should bear more careful considerations on the current landscape of care and patient travel burden. In a simulation study from the United States, regionalization of CHS results in more high-volume centers with only modest increase in patient travel burden (26). This is because many centers performing CHS are geographically clustered in proximity and many patients treated at high-volume centers are already overcoming great travel burden (24). In our study, we noted significant disparities regarding geographic access to care facilities for CHS between different regions, and over 70% of patients from central and western regions needed to travel >180 km to receive high-quality care at facilities with full capacity. The negative impacts of increased travel burden are manifolds. First, the family can suffer heavy financial burden associated with non-medical expenditure for hospitalization or routine clinic visit. Second, geographic barriers may increase the risk of discontinuation of care after surgical treatment, especially during the transition from pediatric to adult service among adolescents and young adults. The importance of geographic barriers is demonstrated by a multicenter study from the United States, which found that the location of heart centers is a strong predictor of gaps in receiving cardiac care among adult CHD patients (27). Under some unusual scenarios, such as the recent outbreak of COVID-19 in China and other countries around the world, the operation of surgical programs for CHD could be severely affected (28). These patients have specialized healthcare needs and may have greater difficulties to receive care due to geographic barriers. In short, the significance of geographic factors on patient travel burden should be emphasized when establishing a regionalized network of CHD care.

### Study Limitations

Our study has several important limitations. First, our study did not analyze the CHS status in the whole nation, nor did we capture all CHS cases in northern China. Nonetheless, the data used in this study are based on annual surveys conducted by the largest academic society of extracorporeal circulation in China and represent the most inclusive data source for cardiac surgery to date. Second, the data source used in this study includes only institution-level data on surgical volume and does not contain detailed information on surgical patient demographics, procedure complexity, mortality, and morbidity in each center, which prevents comparison of surgical outcomes and follow-up of patients from childhood to adulthood for long-term prognosis. In the future, a national registry to capture all relevant CHS cases could provide more comprehensive and detailed epidemiological analysis. Third, facility-level data only reflected the condition at the time when data were collected, and therefore, any subsequent changes were not captured. However, this is a common limitation of all cross-sectional analysis in health service research, and our data are the most updated when collected for analysis.

Designing the ideal care system for children with CHD in China should start from clinical data collection in each center and establishing co-operative research programs that allow for clinical resource integration between different regions. On this basis, future studies can incorporate information on disease burden, treatment capacity, travel distance, and time cost, which provide more in-depth analysis on resource allocation and can help to determine the optimal number and location of surgical programs for CHD. Although such analysis is not possible for the moment due to lack of data, fortunately, relevant stakeholders in China had already began such processes to reach these goals. Our study provides a starting point for such discussion, and more studies are warranted to add more information that can inform policy change in the future.

## Conclusions

In summary, we analyzed the current landscape of care facilities for CHS in northern China and estimated population-based geographic access to these specialist resources. Our results suggest that most facilities for CHS in northern China had limited specialist capacity, and many CHD patients in northern China were receiving suboptimal surgical care for their heart conditions. For many patients from the central and western regions, substantial geographic barriers need to be overcome to receive quality surgical care. Our study provides important information for the discussion about service improvement initiatives and regionalization of specialist resources for CHD, which aims to address the rising burden of CHD in China.

## Data Availability Statement

The raw data supporting the conclusions of this article will be made available by the authors, without undue reservation.

## Ethics Statement

Ethical review and approval was not required for the study on human participants in accordance with the local legislation and institutional requirements. Written informed consent from the participants' legal guardian/next of kin was not required to participate in this study in accordance with the national legislation and the institutional requirements.

## Author Contributions

ZS and HZ conceived the study. ZS, LX, ZL, and HW collected the data. ZS performed the analysis and drafted the manuscript. HZ, SL, and HC provided important revisions for the manuscript. All authors approved the final contents in the manuscript.

## Conflict of Interest

The authors declare that the research was conducted in the absence of any commercial or financial relationships that could be construed as a potential conflict of interest.
